# Estrogen related receptor is required for the testicular development and for the normal sperm axoneme/mitochondrial derivatives in *Drosophila* males

**DOI:** 10.1038/srep40372

**Published:** 2017-01-17

**Authors:** Snigdha Misra, Anuj Kumar Pandey, Snigdha Gupta, Ajay Kumar, Priyanka Khanna, Jai shankar, Kristipati Ravi Ram

**Affiliations:** 1 Embryotoxicology Laboratory, Environmental Toxicology Group, CSIR-Indian Institute of Toxicology Research (CSIR-IITR), MG Marg, Lucknow, Uttar Pradesh, 226001, India; 2 Academy of Scientific and Innovative Research (AcSIR), CSIR-IITR campus, Lucknow, Uttar Pradesh, 226001, India; 3 Electron Microscopy Facility, CSIR-Indian Institute of Toxicology Research (CSIR-IITR), MG Marg, Lucknow, Uttar Pradesh, 226001, India

## Abstract

Estrogen related receptors (ERRs), categorized as orphan nuclear receptors, are critical for energy homeostasis and somatic development. However, significance of ERRs in the development of reproductive organs/organelles/cells remain poorly understood, albeit their homology to estrogen receptors. In this context, here, we show that knockdown of ERR in the testes leads to improperly developed testes with mis-regulation of genes (*aly, mia, bruce, bam, bgcn, fzo and eya*) involved in spermatogenesis, resulting in reduced male fertility. The observed testicular deformity is consistent with the down-regulation of SOX-E group of gene (SOX100B) in Drosophila. We also show dispersion/disintegration of fusomes (microtubule based structures associated with endoplasmic reticulum derived vesicle, interconnecting spermatocytes) in ERR knockdown testes. A few ERR knockdown testes go through spermatogenesis but have significantly fewer sperm. Moreover, flagella of these sperm are defective with abnormal axoneme and severely reduced mitochondrial derivatives, suggesting a possible role for ERR in mitochondrial biogenesis, analogous to mammalian ERRα. Interestingly, similar knockdown of remaining seventeen nuclear receptors did not yield a detectable reproductive or developmental defect in Drosophila. These findings add newer dimensions to the functions envisaged for ERR and provide the foundation for deciphering the relevance of orphan nuclear receptors in ciliopathies and testicular dysgenesis.

Nuclear receptors (NRs) generally function as ligand-regulated transcription factors to modulate a broad range of biological processes as diverse as reproduction, development and metabolism. In higher organisms including human, several such NRs, which associate with hormones and their intermediates, or the orphan receptors that act independent of endocrine ligands have been identified^1^. In the context of reproduction, the roles of estrogen receptors and androgen receptors, which belong to the steroid super family of NRs have been extensively characterized over the decades^2^. Estrogen Related Receptors (ERR), which are constitutively active and whose activity is independent of hormonal ligands^3^,^4^ belong to the same family (NR3) as Estrogen Receptors. Three ERR isoforms (ERRα, ERRβ, and ERRγ) constitute a subfamily (NR3a) of orphan nuclear receptors that share significant amino acid homology with the estrogen receptors ERα and ERβ^5^. Role of ERR has been well documented in placental development and control of lipid metabolism in higher organisms^6^. In addition, ERR is predicted to influence estrogen responsiveness by modulating not only the levels of estrogens but also the expression regime of estrogen-regulated genes in their target tissues^7^,^8^. However, little is known about the involvement of ERR in reproduction across animal kingdom. Recently, our lab reported altered expression as well as activity of ERR in the testes of a lower invertebrate, *Drosophila melanogaster*, in response to a xenobiotic^9^ and provided the first indication for the possible involvement of ERR in male fertility. Subsequently, Yu and co-workers^10^ associated ERR with male fertility based on their genome wide association study of non-obstructive azoospermia in Chinese men coupled with functional screening in Drosophila, and reported the requirement for dERR in testicular development. Beyond this, not much is known about the ERR mediated processes in testicular development as well as spermatogenesis which are critical for male fertility. Therefore, in the present study, an attempt was made to further characterize the role of ERR in male fertility using *Drosophila melanogaster* as a model.

*Drosophila melanogaster*, with its short life-cycle, easily manipulable genome, the repertoire of molecular tools/reagents and the extent of homology of genes associated with testicular development, spermatogenesis, sperm maturation and conservation of protein classes in seminal fluids from insects to mammals, offers an excellent model for the functional characterization of genes essential for male fertility (reviewed in Tiwari *et al*.^11^). Besides, former Drosophila based functional studies with fly homologs of mammalian reproductive genes (including boule) have impressively aided in characterizing the reproductive functions of mammalian counter parts^12^,^13^. Moreover, all the three members [ERRα (NR3B1), ERRβ (NR3B2) and ERRγ (NR3B3)], are represented by a single member, dERR in Drosophila genome^14^. In Drosophila, ERR is involved in major cellular energy homeostasis pathway, and the dERR mutants die as second instar larvae^15^. In this study, by using GAL4 drivers previously established/characterized in the field, we have systematically knocked down dERR through RNA interference (RNAi) across male reproductive tract, in a tissue specific manner, and evaluated the role for ERR, if any, in the development of the male reproductive tract and spermatogenesis. Here, we show that loss of ERR results in improper development of testes and reduced mitochondrial derivatives in sperm axoneme. In addition, we report significantly reduced SOX100B (Sry-related HMG box E homolog critical for testicular morphogenesis^16^) levels and alterations in expression as well as localization of candidate gene products associated with spermatogenesis in response to ERR deficiency. These findings suggest that ERR is not only required for normal testicular development but also necessary for the normal testicular function. These findings suggest that ERR can be used as a molecular handle to address ciliopathic disorders associated with male fertility.

## Results

### Normal Estrogen related receptor levels in different cell types of testes are essential for male fertility in Drosophila

**Figure 1:**
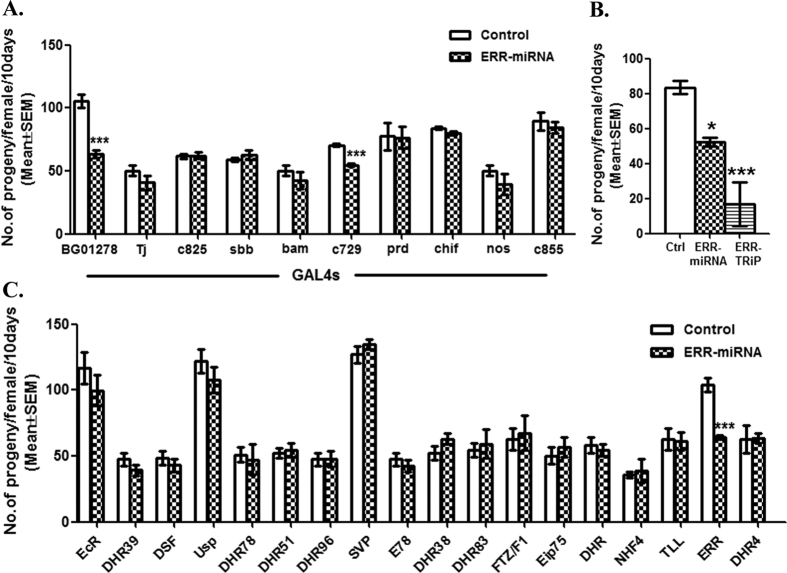
Normal levels of ERR in multiple cell types are essential for male fertility in Drosophila. (**A**) Tissue specific knockdown of ERR mediated by varied male reproductive tract/spermatocytic stage specific GAL4 drivers [Gene trap lines or Prd-GAL4 from Bloomington stock center, USA]. Knockdown of ERR in late spermatogonia, early spermatocytes, cyst cells, pigment cells, seminal vesicles and muscle sheath of the testicular lobes caused significant reduction in the fertility of females mated to knock down males derived from two of the GAL4s: BG01278 (***p < 0.001; N = 15–20; BG01278 bar) and c729 (p < 0.001; N = 15–20; c729 bar). On the contrary, females mated to males knockdown for ERR in tissues other than above (through other GAL4s; bars- c855, c825, sbb, chif, bam, Tj, and nos-GAL4s) or in the accessory glands (mediated through Prd-GAL4; bar prd) had fertility at levels comparable to their controls. (**B**) Females mated to ERR knockdown males (produced from UAS-ERR-miRNA, driven by testes-GAL4) had significant reduction in the number of progeny produced over the span of 10 days (*p < 0.05; represented by ERR-miRNA bar). Similar but robust reduction in the fertility of females mated to ERR knockdown males produced from UAS-ERR-TRiP (***p < 0.001; represented by ERR-TRiP) was observed. (**C**) Eighteen nuclear receptors are known in *Drosophila melanogaster* and knock down of 17 Nuclear Receptors (other than ERR) in a testes-GAL4 specific manner had no significant effect on the male fertility. Females mated to these knockdown males (except those mated to ERR knockdown males; p < 0.001; N = 15–20) had fertility at levels similar to their controls with no significant reduction in the number of progeny produced. This rules out the strain/background effect of nuclear receptor line used on the observed fertility phenotype.

Fertility assays involving males knockdown for ERR in different tissues of their reproductive tract revealed that BG01278-GAL4 (BL12608, hereafter referred to as testes-GAL4) driven ERR knockdown in late spermatogonia, early spermatocytes, cyst cells, pigment cells, muscle sheath of the testicular lobes and/or seminal vesicles caused significant reduction (Fig. 1A; p < 0.001) in the fertility of females mated to knock down males. Similarly, c729-GAL4 (BL6983) based ERR knockdown in male accessory glands, testes sheath and cyst cells also resulted in reduced fertility and is consistent with the reduced fertility observed by Yu and coworkers^10^ with this line. Interestingly, knockdown of ERR in male accessory glands alone (prd-GAL4; BL1947) or in male accessory glands as well as seminal vesicles (c825-GAL4; BL6987) or transiently in early spermatocytes, cyst cells, pigment cells and muscle sheath (sbb-GAL4; BL12772) or in male accessory glands, cyst cells and germ cells (c855-GAL4; BL6989) or in early germ cells (nos-GAL4), or in spermatogonia alone (bam-GAL4) or in early cyst cells alone (Tj-GAL4) did not produce any significant effect on the fertility of females mated to these ERR knockdown males (Fig. 1A). From the above data, it is clear that the male fertility is reduced only when ERR is knocked down simultaneously in both testicular sheath and cyst cells (combination of which are targeted both in testes-GAL4 and c729-GAL4). In addition, transient knockdown of ERR in these cell types has no significant effect on male fertility and the observed reduction in male fertility is independent of other cell/tissue types. These observations suggest the requirement for normal levels of ERR in both testicular sheath and cyst cells for normal male fertility in Drosophila.

Based on the above, subsequent studies were performed by driving the knockdown with testes-GAL4 and two independent RNAi lines (Please see Fig. S1 for the confirmation and the extent of ERR knockdown as observed through qPCR analysis). Females mated to testes-GAL4 driven ERR knockdown males produced significantly fewer progeny (p < 0.05; Fig. 1B; ERR-miRNA bar), when compared to those of control mates (Fig. 1B; control bar). Similar but even more robust fertility phenotype was observed with ERR-TRiP knockdown males: except for one or two replicates, majority of the females mated to ERR- TRiP males did not produce any progeny (p < 0.001; Fig. 1B; ERR-TRiP). In contrast, similar knockdown of remaining seventeen nuclear receptors, individually, had no significant effect on the male fertility: females mated to these knockdown males had fertility at levels similar to controls (Fig. 1C). This lack of phenotype with the remaining lines argues against the possibility of the strain background influencing the observed fertility reduction in ERR knockdown males. These data suggest that ERR is one of the most vital nuclear receptor for normal male fertility in Drosophila.

### ERR knockdown males have deformed testes and significantly reduced Sox100B levels in their gonads

**Figure 2:**
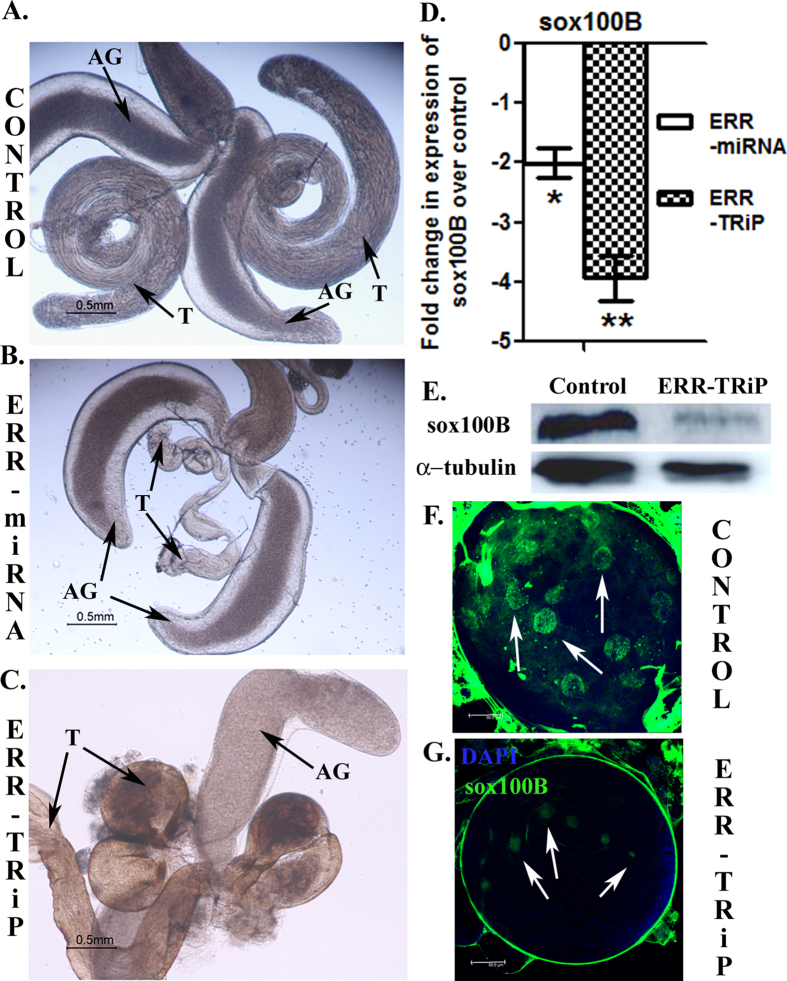
Testicular deformity in ERR deficiency is consistent with the reduced levels of, *sox100B* in the adult testes as well as larval gonads of ERR knockdown males. (**A**) The male reproductive tract of control male, with elongated testes (T) and normal accessory glands (AG). (**B**) Underdeveloped testes in ERR knockdown males produced from ERR-miRNA. (**C**) Extremely deformed and globular testes in ERR knockdown males produced from ERR-TRiP line. (**D**) The levels of Sox100B transcripts were reduced to 2 folds in testes of ERR-miRNA knockdown males (*p < 0.05, represented as ERR-miRNA) while 4 folds reduction was evident in ERR-TRiP knockdown males (**p < 0.01, represented as ERR-TRiP). The ∆ct values were determined through normalization against Act-5c, which was used as an internal control for the quality of the template. (**E**) The adult testes of ERR knocked down males, probed with Sox100B antibody reflected marked decrease in the levels of Sox100B protein when compared to their respective control (Panel Sox100B); α-tubulin served as loading control (Panel α-tubulin). (**F**) Immunofluorescence based detection of Sox100B in larval male gonad revealed higher Sox100B positive signals (green dots marked by white arrows) in the central transparent region of control male gonad and (**G**) Very few Sox100B positive cells with relatively weaker signal intensity (faint green dots, marked by white arrows) in ERR knockdown (ERR-TRiP) male gonads. The panels represent the overlay of nuclear (DAPI in blue) and Sox100B (green) staining.

Males with normal levels of ERR had morphologically normal reproductive tract (Fig. 2A). On the contrary, the knockdown males produced from both the ERR lines had partially/completely underdeveloped testicular lobes and seminal vesicles (Fig. 2B and C respectively), consistent with the report of Yu and coworkers^10^. However, the frequency of testicular deformity phenotype was variable with only 20% (10 males out of 50 dissected) in knockdown males produced from ERR-miRNA line while it was 90% in the knockdown males produced from ERR-TRiP line (45 males out of the total 50 knockdown males dissected had the deformed testes). In addition to the frequency of the testicular deformity, the intensity and robustness of the phenotype was also severe in knockdown males, produced from ERR-TRiP line. In these knockdown males, the testicular lobes existed as globular masses. The observed differences in the frequency of testicular deformity between the two RNAi lines can be attributed to the magnitude of knockdown of ERR in these lines (Fig. S1). The remaining tissues of the reproductive tract appeared to be same as in controls.

SOX100B mutants exhibit a very similar phenotype of failed gonadal morphogenesis^16^, as observed with ERR mutants. Besides, SOX100B is a fly orthologue of SOX9, a Sox domain transcription factor, required for testis development in mammals^17^,^18^. Since the testis was abnormal in ERR knockdown males, the expression pattern/localization of Sox100B were subsequently determined in ERR knockdown males. The ERR knockdown males produced from both the ERR lines exhibited significant 2–4 fold down regulation (*p < 0.05, **p < 0.01; Fig. 2D) in the testicular transcript levels of *sox100B*, when compared to those in the genetically matched control males. Consistent with this, we observed reduced Sox100B protein levels in ERR knockdown males, when compared to their respective controls (Fig. 2E). Analysis of localization of Sox100B in the male gonads from ERR knockdown (ERR-TRiP)/control larvae (early III instar) revealed relatively higher Sox100B positive cells in the central region of the transparent control gonads (Fig. 2F; Sox100B positive cells indicated by white arrows) when stained with Sox100B primary antibody, detected with Alexa 488 secondary antibody (please refer to Table S3 in supplementary material for additional details). On the contrary, ERR knockdown (ERR-TRiP) male gonads were small and had either no Sox100B positive cells, or very few such cells with weaker signal intensity (Fig. 2G; Sox100B signals marked by arrows).

### Significant alterations in the transcript levels of genes associated with spermatogenesis in the ERR knockdown males

**Figure 3:**
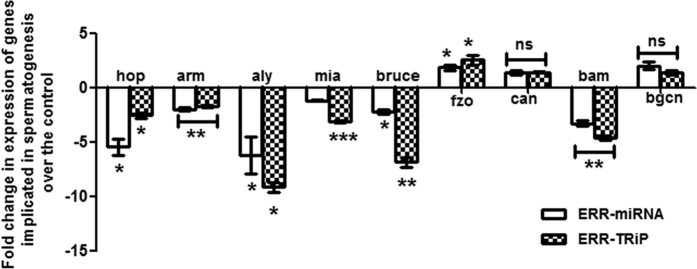
Mis-regulation of certain genes implicated in the process of spermatogenesis in testes from ERR knockdown males. The transcript levels of *hop, arm, aly, mia, bruce, and bam,* were significantly down-regulated in ERR knockdown male testes (either from ERR-miRNA or ERR-TRiP). The transcript levels of *can* and *bgcn* in knockdown males from both lines did not differ from those in control. The transcript levels of *fzo* were significantly upregulated. The ∆ct values were determined through normalization against Act-5c, which was used as an internal control for the quality of the template. The significance levels have been represented in terms of *p < 0.05, **p < 0.01, ***p < 0.001.

We analyzed the transcript levels of nine genes, involved in the process of spermatogenesis, through quantitative real-time PCR. The expression levels of six genes, namely, *hop, arm, aly, mia, bruce*, and *bam,* were significantly reduced (*p < 0.05; **p < 0.01, and ***p < 0.001; Fig. 3) in testes from ERR knockdown males when compared to those in genetically matched controls. However, transcript levels of *can* and *bgcn* were similar (represented as ns; Fig. 3) and levels of *fzo* were significantly upregulated (*p < 0.05; Fig. 3) compared to those in controls (represented as baseline in Fig. 3). In addition, the observed trend of up/down-regulation of genes was consistent between knockdown males, generated from two different lines (ERR-miRNA, and ERR-TRiP).

Since the frequency of deformity was much higher (90%) in the knockdown males produced from the ERR-TRiP line, experiments involving immunolocalization of germ and cyst cell markers in testes, sperm production and sperm storage were performed using these knockdown males (testes-GAL4/ERR-TRiP) and their genetically matched controls (Sb/ERR-TRiP).

### Loss of ERR disrupts the germ and cyst cells in Drosophila testes

**Figure 4:**
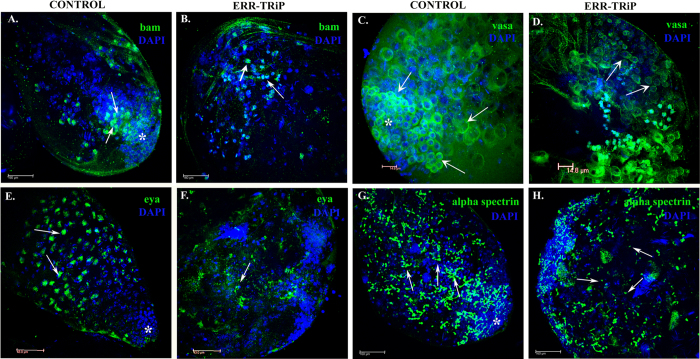
Localization of germ/cyst cell marker protein in the ERR knockdown/control male testes. To detect the effect of ERR knockdown on germ/cyst cell lineages in Drosophila testes, we analyzed the localization of bag of marbles (bam; (**A,B**) in green), vasa (**C,D**) in green) eyes absent (eya; (**E,F**) in green) and alpha-spectrin ((**G,H**), in green) in the control and knockdown testes through immunofluorescence. DAPI (blue) was used to stain the nuclei of the cells. We detected bam in marking the germ cell/spermatogonia in control testes (Panel A; green in CONTROL) but very randomly localised/feeble signals were observed in ERR knockdown testes (Panel B; green in ERR-TRiP). Similarly vasa marking the germ cells were prominently evident in control testes (Panel C; green in CONTROL) showing compactly arranged germ cells underneath the hub, and loosely, yet uniformly scattered in tissue apex. No such discrete pattern could be seen in knockdown testes (Panel D; green in ERR-TRiP) where germ cells marked, were randomly arranged. Eya marking the late somatic cyst cells were optimally evident in control testes (green, Panel E) but very reduced number as well as intensity of eya signals were visible in ERR knockdown testes (Panel F; green in ERR-TRiP) despite the presence of cells as evident in the nuclear staining of the same, through DAPI (blue signals). Similarly, in control testes, alpha-spectrin marked profusely branched fusomes (green; Panel G), indicative of the normal 8–16 celled secondary spermatocytes. In contrast ERR knockdown testes had a very few branched fusomes/more linear and small fusomes (Panel H; ERR-TRiP) as marked by alpha-spectrin.

The ERR knockdown males were probed for the germ [bam (bag of marbles) and vasa, expressed in spermatogonia and germline respectively] and cyst cell markers [eya (eyes absent), expressed in somatic cyst cells] to determine the effect of ERR deficiency on the cell types. In testes from the genetically matched control males, bam was localized (Fig. 4A, bam indicated in green) in the cells just beneath the testicular apex, indicating the spermatogonial cells. In contrast, ERR knockdown testes (ERR-TRiP) had feeble/fewer detectable bam staining/signal (Fig. 4B). This is consistent with our real-time PCR observations of reduced bam transcript levels in ERR knockdown testes. Similarly, in controls, all the germline cells in their varied stages (from small spermatogonial cells to large spermatocytes) were uniformly evident, all along the tissue apex prominently marked by the vasa (Fig. 4C; vasa; indicated in green). In contrast, vasa signals were fewer and randomly arranged with no distinct pattern/arrangement (Fig. 4D; vasa; indicated in green) of germ cells in ERR knockdown testes. The somatic cyst cells were probed with eya which was again found to be uniformly distributed, marking the nuclei of somatic cyst cells, enclosing the late spermatocytes, located underneath the region flanked by spermatogonial group of cells at the tip of testes (Fig. 4E. eya indicated by green signals). However, in testes from ERR knockdown (ERR-TRiP) males, eya was markedly reduced (Fig. 4F) unlike their optimal number/synchronised pattern, as evident in controls. The fusome (an organelle comprising microtubules presumably derived from endoplasmic reticulum) serving as interconnections between the adjoining germ cells^19^, being exhibited by the alpha-spectrin staining in adult testes (Fig. 4G), was found to be highly branched in control testes (indicated by the white arrow) in the region of spermatocytes, indicating the presence of considerably higher number of cells (8–16) within spermatocytes. Interestingly, such branching of fusomes was limited in ERR knockdown (ERR-TRiP) testes (Fig. 4H), instead, several linear fusomes (indicated by the white arrow) appeared in staining, suggestive of fewer cells being held together in late spermatocytic stages.

### ERR knockdown males contain significantly fewer mature sperm in their seminal vesicles

**Figure 5:**
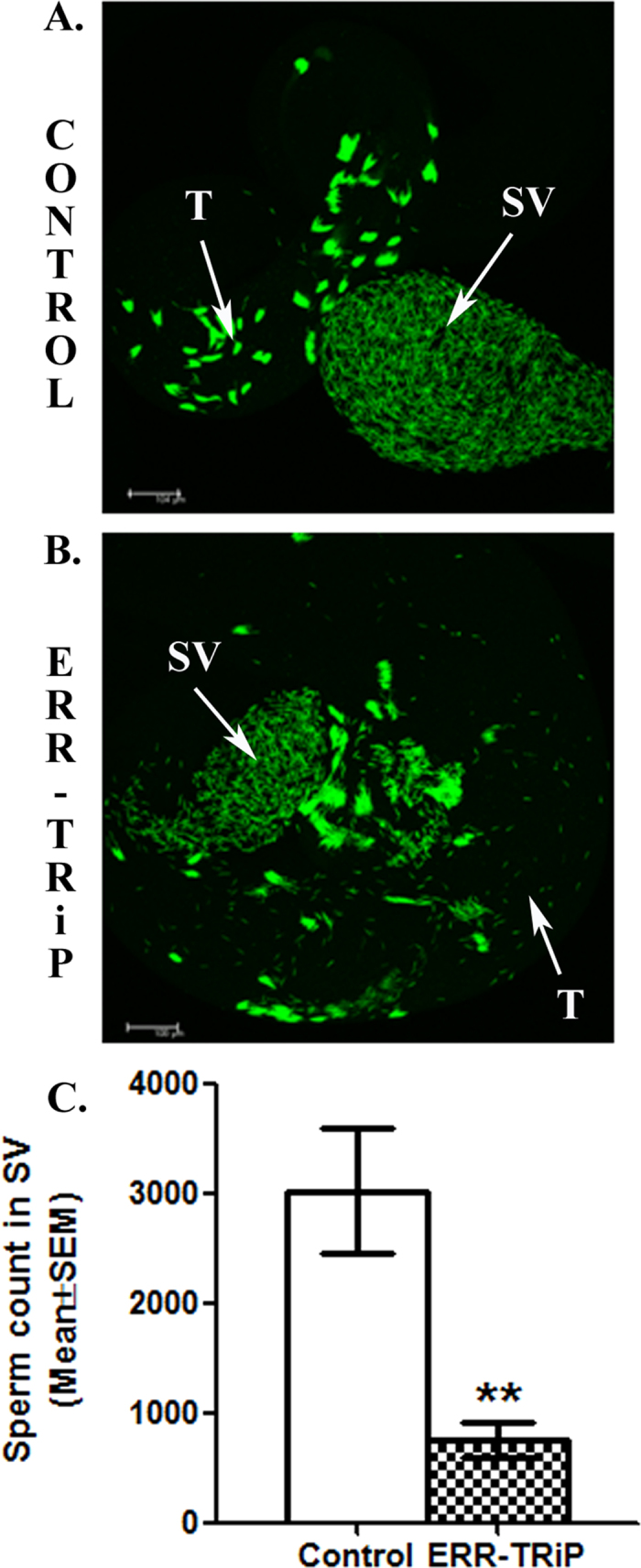
Fewer mature sperm in the testes of ERR knockdown males dispersed all along the testicular tract. To determine if the impact of ERR on molecular players associated with spermatogenesis is reflected at the phenotypic level, we analyzed Protamine incorporation into sperm chromatin in control/knockdown males carrying ProtamineB-EGFP and counted the number of mature sperm. Both control and knockdown testes/seminal vesicles displayed sperm labeled with GFP (Green, Panels A and B) suggesting normal protamine incorporation. Further, control males (Panel A) have mature sperm in distended seminal vesicle (SV; indicated by white arrow). However, knockdown males had sperm (in green) scattered all along the globular testicular mass (Panel B, ERR-TRiP, labeled as T). The number of mature sperm in the SV (Panel C) of ERR knockdown males were significantly reduced (**p < 0.01). All these experiments were carried out twice and sperm were counted in tissues twice with a repeatability of index of 92%.

To investigate whether the exchange of histones for protamines is normal in spermatids from ERR knockdown flies, we examined spermatid nuclei of ERR control/knockdown males carrying a protamineB-EGFP construct. We observed that spermatid nuclear bundles and nuclei of individualized sperm show a clear GFP signal in control as well as knockdown testes (Fig. 5A and B; green), which suggests that incorporation of protamines into sperm chromatin is independent of ERR. Subsequently, the sperm stored in seminal vesicles of ERR knockdown males (ERR-TRiP) and their genetically matched controls were counted. The control males contained mature sperm in their seminal vesicles (Fig. 5A). On the contrary, the knockdown males (ERR-TRiP), having deformed testes, exhibited mature sperm dispersed all through the deformed globular testes (Fig. 5B). In addition, the number of mature sperm stored in the seminal vesicles of ERR knockdown males were significantly reduced when compared to that in the seminal vesicles of controls (**p < 0.01; Fig. 5C).

**Figure 6:**
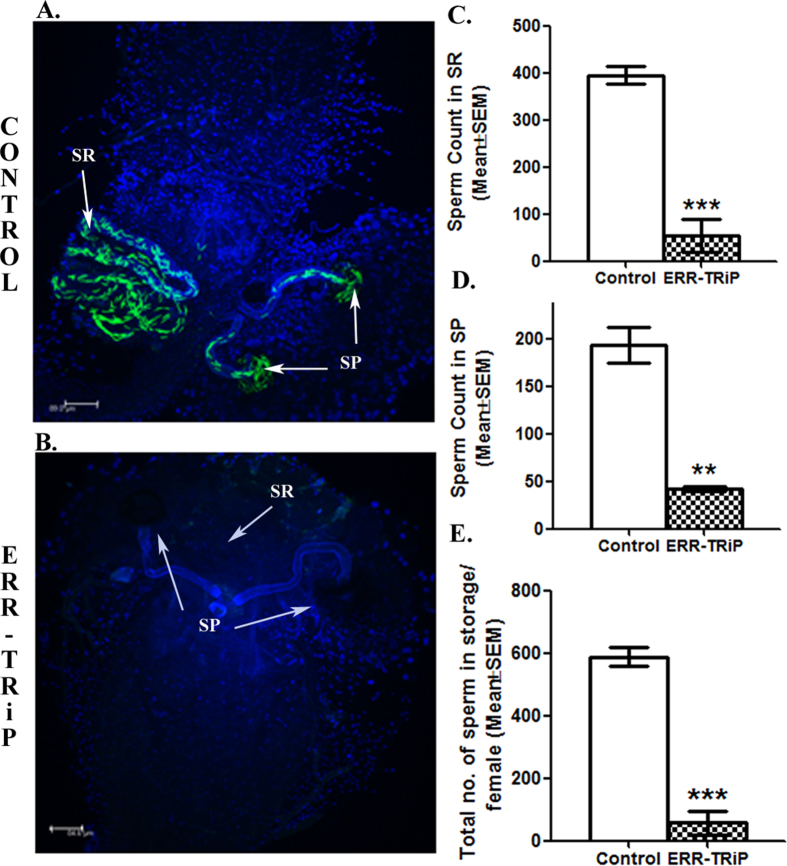
Storage of sperm in females mated to ERR control/knockdown males. (**A**) Sperm storage in seminal receptacle (green signals in SR; marked by white arrow) and spermathecae (green signals in SP; marked by white arrow) of female mated to genetically matched control males. (**B**) Sperm storage in SR and SP of female mated to ERR knockdown (ERR-TRiP) male having globular testes. No sperm storage could be seen in the SR and SP region (marked by white arrows) of the female tract, indicating the reason of complete sterility exhibited by 90% of them. The remaining females mated to ERR knockdown males with visibly normal testes had extremely fewer sperm in their SR (Panel C; ***p < 0.001) and SP (Panel D; **p < 0.01) reflecting significantly fewer sperm in their storage (total, Panel E; ***p < 0.001) at 2 h ASM, when compared to their controls.

Despite the formation of sperm in ERR knockdown male tracts, 90% of females mated to such males were sterile. Therefore, to determine the level of sperm storage in the mated females, ERR knockdown (ERR-TRiP)/control males were mated to 3–5 days old Oregon-R virgin females. On dissecting the reproductive tracts of mates of control males, storage of sperm in their sperm storage organs, seminal receptacle (green signals in SR; Fig. 6A) and spermathecae (green in SP; Fig. 6A) was evident. In contrast, reproductive tracts of females mated to ERR knockdown (ERR-TRiP) males with testicular deformity (globular testes), did not have sperm in their storage (Fig. 6B). However, females mated to those ERR knockdown males (ERR-TRiP), which exhibited phenotypically normal tract, had sperm in their storage but still significantly reduced (seminal receptacle, ***p < 0.001, Fig. 6C; spermathecae, **p < 0.01, Fig. 6D; total sperm in storage; ***p < 0.001, Fig. 6E) when compared to the sperm storage levels in mates of control males. Therefore, the observed reduction in fertility of ERR knockdown (ERR-TRiP) mates is a consequence of lack of sperm transfer or the reduced storage of transferred sperm.

### Disorganized sperm bundles and abnormal sperm axonemes in testes from ERR knockdown males

**Figure 7:**
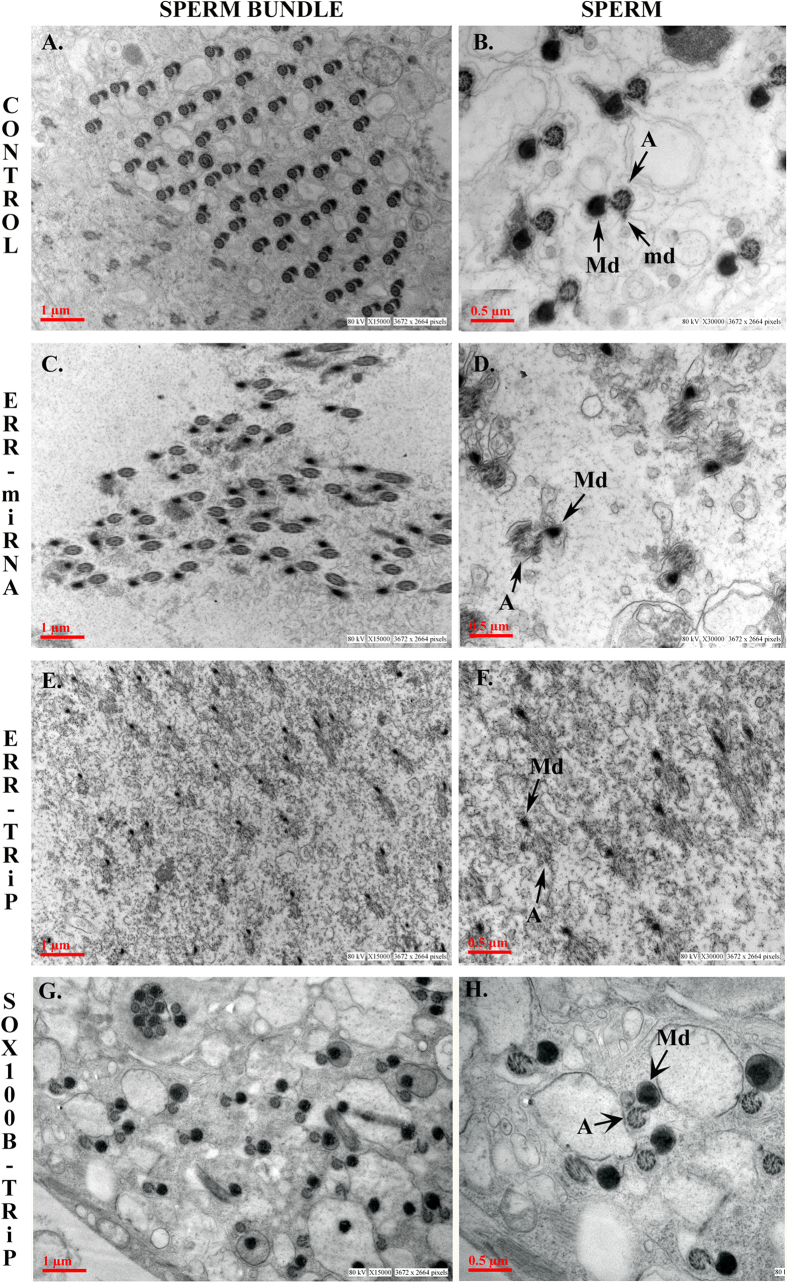
Reduced mitochondrial derivatives in the sperm flagella of ERR knockdown males, and nicked/defective axonemes in the sperm flagella of Sox100B knockdown males. Ultrastructural analysis of sperm bundles, and sperm axoneme of ERR control/knockdown males through transmission electron microscopy revealed the perfect alignment of the spermatids in the sperm bundles of control males’ testes (Panel A). However, this arrangement is completely disrupted in the ERR knockdown males (Panel C; ERR-miRNA, and Panel E; ERR-TRiP) and random in Sox100B knockdown pupal testes (Panel G; Sox100B-TRiP). Panel B depicts the cross-sections of the sperm axonemes with a characteristic 9 × 2 + 2 pattern of the axoneme (represented by A), the electron dense, major (represented by Md) and minor (represented by md) mitochondrial derivatives in control testes. Panel D (ERR-miRNA), and Panel F (ERR-TRiP) represent the sperm axonemes in ERR knockdown males with clearly disrupted 9 × 2 + 2 arrangement, in addition to highly reduced Md and remnant md. Similarly, Panel H (Sox100B-TRiP) represents the nicked sperm axoneme lacking the complete circular shape of the microtubular spoke but normal mitochondrial derivatives in Sox100B knockdown sperm axoneme.

To determine changes, if any, at the structural level in the testes due to the deficiency of ERR, testes of knockdown males generated from both ERR lines, and their controls were observed under the transmission electron microscope. Electron-micrographs revealed that the control males had perfect alignment of the spermatids in the sperm bundles, with each bundle clearly demarcated from the neighbouring bundles (Fig. 7A). In addition, in the sperm flagellum, the major and minor mitochondrial derivatives, associated with axonemes were clearly visible in controls (Fig. 7B) while microtubules showed a characteristic 9 × 2 + 2 pattern (Fig. 7B). On the contrary, testes from the ERR knockdown males (ERR-miRNA or ERR-TRiP) had abnormal sperm bundles, many of which had fused with the neighbouring bundles (Fig. 7C and E). Further, the major mitochondrial derivative was observed to be markedly reduced, appearing in the form of small dark peck and the 9 × 2 + 2 arrangement of the microtubule was also disrupted (Fig. 7D and F) to severe levels in the knockdown males generated from the two lines.

### Sperm from Sox100B knockdown males contain normal mitochondrial derivatives but abnormal axonemes

To assess if the observed sperm defects are a consequence of the defective testicular morphogenesis, we examined whether Sox100B RNAi males (with deformed testes) have similar sperm defects. Since Sox100B RNAi leads to pupal mortality (similar to that reported in Sox100B mutants^16^), we analyzed pupal testes under transmission electron microscope. The transmission electron micrographs of testes from Sox100B RNAi males revealed defects in sperm flagellar structure, albeit less severe and importantly, different from those observed in sperm from ERR knockdown males. Unlike the reduced mitochondria in the ERR knockdown sperm, the mitochondrial derivatives in Sox100B knockdown sperm were comparable to those in control (Fig. 7G). However, certain axoneme exhibited evident nick in the 9 × 2 + 2 arrangement of the microtubule towards the major mitochondrial derivative junction (Fig. 7H). Additionally, we examined the levels of ERR transcripts in Sox100B knocked down male pupae through quantitative real-time PCR. We observed that the levels of ERR are comparable to those in controls. (Please see Fig. S2).

## Discussion

Understanding the role of ERR in reproduction was essential as majority of the reports implicated ERR in somatic functions^6^,^15^,^20^,^21^,^22^,^23^,^24^,^25^,^26^,^27^ and limited knowledge exists on the reproductive roles of ERR. Earlier, Yu and co-workers^10^ in single nucleotide polymorphism based screen, associated ERR with male fertility and reported a role for dERR in testicular morphogenesis. To further characterize the role of ERR in male fertility, in the current study, we knocked down ERR in a tissue/cell specific manner in the Drosophila male reproductive tract and examined the effect of ERR deficiency on candidates associated with testicular morphogenesis or spermatogenesis at the transcript and biochemical levels. Our observation of significantly reduced fertility of females mated to males knockdown for ERR and testicular deformity in ERR knockdown males support the findings of Yu *et al*.^10^ that ERR is essential for male fertility due to requirement for ERR in testicular morphogenesis. In addition, our cell/tissue-type based knockdown study suggests that the reduced fertility is a consequence of the role(s) for ERR in both testicular sheath and cyst cells. Together, these data suggest that Drosophila can offer an excellent model to understand the reproductive roles of ERR.

Identification of role(s) for ERR in testicular morphogenesis can have potential ramifications for spermatogenesis. Consistent with this, in the present study, genes associated with spermatogenesis (*bam, hop, arm, aly, mia, bruce and fzo*) were found to be mis-regulated in the ERR knockdown testes. *Bam* is required for the differentiation of germline stem cell daughters and their progress beyond the cystoblast^28^. In the absence of bam, germ cells become trapped as mitotically active non-differentiating cells^28^. *Hop* is associated with discrepancies in meiotic divisions, that result in failure of flagellum formation, generating sperm tail defects, affecting their motility^29^. *Arm* is known to regulate normal differentiation in *Drosophila* male gonad^30^. In addition, ‘meiotic arrest’ class of genes, always early (*aly*) and meiotic arrest (*mia*) are required for spermatid differentiation^31^, while *bruce* is required for protection of the sperm nucleus against hypercondensation and degeneration at the time of spermatid terminal differentiation and individualization^32^. Likewise, fuzzy onion (*fzo*), is required for controlled mitochondrial fusion during Drosophila spermatogenesis^33^,^34^,^35^,^36^,^37^ and is required for the formation of the two giant mitochondria that comprise the mitochondrial derivative (Nebenkern^38^) in individual sperm. In mammals, ERR is known to be a transcriptional activator^39^ and observed mis-regulation of above genes in the present study may be a consequence of this. Alternatively, ERR knockdown may have caused a relative depletion of a spermatogenic stage in which particular transcript (s) is/are abundant, leading to observed reduction in transcript levels in whole-testes as a simple consequence of there being fewer cells at the stage that highly express it. Although we did not characterize the spermatogenic stage distributions by microscopy, we examined a few markers such as bam, vasa, eya, and alpha spectrin through immunofluorescence to mark the germ cells, and the adjoining cyst cells. The observed reduction in the levels and differences in the localization of bam, vasa, eya as well as alpha spectrin, between control and the knockdown testes, reflects the disrupted, germline/somatic cell lineages, with disorganized interconnecting strands/fusomes. Interestingly, the dispersal of fusome contents, which are microtubule based structures associated with ER derived vesicles^19^, has previously been associated with sterility^40^. Based on the above, it is logical to expect that the observed mis- expression/localization of different molecular players in spermatogenesis culminates in reduced sperm production. Consistent with this, the number of mature sperm (with protamine incorporated chromatin) were significantly reduced in ERR knockdown males when compared to those in controls, suggesting that [1] atleast a few of the germ cells could still progress through the spermatogenesis in ERR knockdown background to form mature sperm and that [2] ERR is necessary for normal spermatogenesis. However, further studies are required to elucidate the mechanism and/or molecular players underlying the ERR dependent effects on spermatogenesis.

In higher organisms, estrogen related receptor alpha (ERRα) is known to induce mitochondrial biogenesis in a cell-type dependent manner^41^. In the context of reproduction, mitochondria, housed in the sperm flagella, typically power the sperm in their endeavour to reach the egg. Sperm flagellum typically comprises a central axoneme composed of microtubules with 9 × 2 + 2 arrangement, darkly stained major, and lightly stained minor mitochondrial derivatives^42^. Given the homology of dERR with ERRα and our observation of reduced transfer/storage of sperm from ERR knockdown males, we hypothesized that significant reduction in dERR levels in the knockdown testes would affect the sperm mitochondrial derivatives. To test if this is the case, we examined the sperm from ERR knockdown males at the ultrastructural level through transmission electron microscopy. Consistent with our hypothesis, microtubule arrangement was severely disrupted and mitochondrial derivatives in majority of the sperm axonemes were extremely reduced, indicating that even if the sperm are being formed in ERR knockdown males, majority of these are abnormal, and defective in their flagellar structure. These observations point to a plausible analogous role for ERR in mitochondrial biogenesis even in Drosophila. Therefore, our findings highlight the requirement for ERR in normal spermatogenesis and sperm flagellar formation. The present study does indicate that ERR is not only required in testicular morphogenesis but is also essential for the formation of normal functional gametes essential for male fertility in Drosophila.

The mechanism underlying ERR mediated role(s) in testicular development and/or function appears to be complex. Nuclear receptors regulate their target genes by acting as transcription factors and directly interact (as monomers, homodimers, or heterodimers) with DNA response elements within target genes, as well as through cross-talk with signalling pathways (reviewed in Aranda *et al*.^43^). Lack of a detectable reproductive phenotype when the remaining seventeen NRs (other than ERR) were knocked down using the same GAL4 driver raises the possibility of ERR regulating testicular development as a monomer or homodimer in Drosophila, coherent with the mode of action of orphan nuclear receptors. At this juncture, it is pertinent to note that the deformed testicular phenotype in ERR knockdown male is similar to that observed in Sox100B mutants^16^, wherein failed gonadal morphogenesis tends to keep the gonads in the characteristic oval shape of the larval testis. In addition, as with *Sox* gene mutations^16^,^44^, the testicular deformity observed in ERR knockdown males was quite variable with occasional males having normal testes. Sox100B, Drosophila ortholog of mammalian Sox9, expresses in male-specific somatic gonadal precursors and in pigment cells^45^,^46^. Sox9, a SoxE group of gene required for testis development in mammals, exhibits male-specific gonadal expression in vertebrates^47^,^48^. In mammals, Sox9 has been implicated as a target gene of ERRα in chondrocytes^49^ and over-expression of ERRα positively regulates the mRNA and protein levels of Sox9^50^. The observed reduction of Sox100B levels in ERR knockdown testes in the present study are consistent with the above. In addition, normal levels of ERR transcripts in Sox100B knockdown suggest that Sox100B is down-stream of ERR in Drosophila. The loss of ERR probably leads to the reduction of Sox100B and that in turn may lead to deformed testes in ERR knockdown males. To further understand the role of ERR in sperm flagellum, we asked if the observed sperm defects in ERR knockdown males are a consequence of testicular deformity. If that is the case, one would expect to see similar sperm defects in Sox100B RNAi having deformed testes. Therefore, we examined the deformed testes from Sox100B RNAi pupae at the ultrastructural level for sperm defects. Interestingly, sperm from Sox100B RNAi deformed testes had defective 9 × 2 + 2 microtubule but contained mitochondrial derivatives comparable to those in controls. Therefore, the observed mitochondrial defects in ERR knockdown sperm may not be an ultimate consequence of testicular deformity. These findings point to a plausible role for ERR in mitochondrial biogenesis in Drosophila. The distinct phenotypic differences in the sperm defects observed between ERR and Sox100B RNAi suggest that both these genes are essential for normal sperm axoneme in Drosophila and that these genes might act in independent pathways that regulate microtubule arrangement in the sperm axoneme. These findings suggest that multiple candidates in addition to ERR and Sox100B, might be involved in testicular development and function in Drosophila. In beetle (*Tribolium castaneum*), injection of dsRNA against E75 or DHR39 was shown to reduce sperm production^2^. Therefore, an alternative explanation for the lack of detectable phenotype for the remaining NRs in the present study is that ERR may be a critical candidate and other receptors might play accessory roles, and fertility assay employed in this study might not be sensitive enough to detect the same. Consistent with this notion, knockdown of ecdysone receptor (EcR) signalling in otherwise wild-type testes through various experimental methods had no detectable phenotype but modulation of EcR in an Epidermal growth factor (EGF) signalling mutant background suggested that EcR acts in a pathway opposing EGF for cyst differentiation in Drosophila^51^,^52^. Interestingly, in mammals, EGF signalling selectively activates ERRα target genes^53^. Therefore, future studies on molecular cross-talk involving ERR and Sox100B with EGF signalling would pave the way for understanding the mechanisms underlying ERR role(s) in normal testicular development.

In mammals, knockout of estrogen receptors leads to fluid accumulation within testes, which impairs sperm production and causes male infertility^54^. In this context, observed male reproductive role for ERR in Drosophila, an insect which is very low in the evolutionary hierarchy and lacks estrogen or estrogen receptor homologs^55^ may point to a primitive estrogen- like mechanism defining male fertility in lower organisms. Therefore, this finding raises further concern over the potential adverse effects of environmental estrogens on the male fertility of lower organisms such as insects of economic importance. To conclude, ERR is critical not only for establishing the testicular architecture but also for the structural integrity of sperm flagellum in Drosophila. The present study demonstrates important role(s) for ERR in male fertility and thereby provides a molecular handle to understand the functional significance of orphan nuclear receptors in gonadal dysgenesis in males and to address ciliopathic disorders associated with male fertility.

## Materials and Methods

### Fly strains

We used six publicly available gene trap (pGT1) based GAL4 lines [c855-GAL4 (BL6989), c825-GAL4 (BL6987), sbb-GAL4 (BL12772), chif-GAL4 (BL13134), c729-GAL4 (BL6983), BG01278-GAL4 (BL12608)] previously characterized by Bunt and coworkers^56^ for their expression in testes (please see Table S1 for the expression pattern), in addition to prd-GAL4 (BL1947, male accessory glands), bam-GAL4 (specific to spermatogonia), Tj-GAL4 (early cyst cells alone), and nos-GAL4 (early germ cells) to drive the knockdown of ERR in various regions of the male reproductive tract. To mimic the mutant scenario of Sox100B, dsRNA against the same were driven through Act5c-GAL4/Tb^57^. Transgenic fly line carrying miRNA construct for ERR [w[*]; P{y[+t7.7] w[+mC] = UAS-ERR.miRNA}attP16/CyO (BL44391; designated as ERR-miRNA)] and the other miRNA based NR lines (EcR, DHR39, DSF, USP, DHR78, DHR51, DHR96, SVP, E78, DHR38, DHR83, FTZ/F1, EiP75, DHR, NHF4, TLL, and DHR4) were used as in Lin *et al*.^58^. All the GAL4 lines and additional transgenic lines carrying RNAi construct for ERR [y^1^v^1^;P{TRiP.JF02431}attP2; BL27085; designated as ERR-TRiP], and Sox100B [y^1^sc^*^v^1^;P{TRiP.HMC04723}attP40; BL57417, and y^1^sc^*^v^1^;P{TRiP.GLV21021}attP2; BL35656, designated as Sox100B TRiP] used in the present study, were obtained from Bloomington Drosophila Stock Center (BDSC), USA. The two ERR RNAi lines carry different constructs and target different regions (target sequences separated by at least 200 bp) of ERR transcript. In addition, prediction of off-targets for the concerned dsRNA generating sequences through two independent prediction programs: dscheck^59^ or online OTE (off-target effect) prediction tool of DRSC (www.flyrnai.org) did not yield any potential off-targets. To analyze sperm counts and protamine incorporation into sperm chromatin, GAL4 driver line with Protamine-EGFP tagged sperm (ProtB-EGFP/ProtB-EGFP; BG01278-GAL4/TM3 Sb) was generated by a series of crosses between BG01278-GAL4 and Protamine B-EGFP (X); TM3/TM6^60^. In addition, flies (particularly females) from wild type *D. melanogaster* (Oregon-R) were used to fulfil the requirements of assays of fertility and sperm storage. All flies were reared under a 12:12 h light-dark cycle at 22 ± 1 °C on standard Drosophila maize-sugar food medium supplemented with additional yeast in glass bottles/vials. The experiments included in this paper have been carried out after the research programme has been duly approved by the Research Council of the institute.

### Generation of knockdown and controls

Initially, males knockdown for ERR (in different tissues of the male reproductive tract) and control males were generated by crossing UAS- ERR-miRNA/CyO females to above mentioned GAL4 males. The resultant male progeny with the balancer (Cyo for progeny from crosses involving gene trap lines and Sb for progeny from prd-GAL4/Sb) formed the control groups and the male progeny without the relevant balancer (UAS-ERR-miRNA; GAL4) formed the knockdown group. Based on the outcome of this screen, BG01278-GAL4 or testes-GAL4 was used to generate control (Cyo; testes-GAL4) and knockdown (UAS-ERR-miRNA; testes-GAL4) males in subsequent experiments after initial fertility assessment. While using TRiP line for ERR knockdown, testes-GAL4/TM3 Sb flies were crossed with ERR-TRip flies to generate knockdown (UAS-ERR-TRiP/testes-GAL4) and genetically matched control males (UAS-ERR-TRiP/Sb). Likewise Act5c-GAL4/Tb flies were crossed with Sox100B-TRiP lines to generate knockdown(UAS-Sox100B-TRiP/Act5c-GAL4) and genetically matched control males (UAS-Sox100B-TRiP/Tb). Since systemic knockdown of Sox100B exhibited pupal mortality (like the Sox100B mutants^16^), deformed testes from late pupal stages were used for the study.

### Fertility assay

The reproductive performance of control and knockdown males was assayed by analyzing fertility (number of progeny eclosed) of their mates following Misra *et al*.^9^. Briefly, control/knockdown males were pair-mated, independently, with 3–5 days old wild type Oregon-R virgin females. Matings were observed to ensure copulation and only those matings that lasted 15 mins or more were considered as successful. At the end of mating, males were removed from the vials and females were allowed to lay eggs for 10 days ASM (After the Start of Mating) with transfers to fresh control food vials, every three days. Subsequently, flies emerging in these vials were counted and fertility was represented in terms of the total number of progeny produced over a period of 10 days by females mated to control/knockdown males. The differences in overall fertility were statistically analyzed through one way Analysis of Variance (ANOVA) followed by Tukey’s multiple comparison tests. All these assays were repeated more than three times with each group consisting of minimum of 20 replicates.

### Light microscopy

The reproductive tracts from ERR knockdown males and their controls were dissected and analyzed for malformations, if any, in the male reproductive tracts. Whole reproductive tracts from control and knockdown males were dissected in physiological saline on a slide. The coverslips were placed, sealed with nail polish, and images were captured through an inverted microscope (Nikon, Japan) at a total magnification of 100X. These observations were performed in five independent batches, each having at least 5 replicates (pairs of testes).

### Real-time PCR

The expression levels of the ten candidate genes critical for the process of spermatogenesis [armadillo (*arm*^61^), hopscotch (*hop*^62^), always early (*aly*^63^), meiotic arrest (*mia*^64^), *bruce*^65^, fuzzy onion (*fzo*^36^), cannonball (*can*^66^), bag of marbles (*bam*^67^), benign gonial cell neoplasm (*bgcn*^68^)] and/or testes development (*Sox100b*^16^) were analyzed by real time PCR in both ERR knockdown lines. Similarly, transcript levels of ERR were determined in Sox100B knockdown lines. Total RNA was extracted from 35 pairs of adult testes dissected from control/ERR knockdown males or equal number of pupal testes dissected from control/Sox100B knockdown male pupae. Subsequently, we carried out cDNA synthesis following manufacturers’ protocol (Superscript III, Invitrogen, USA). Real-time PCR (qPCR) was carried out as described in Misra *et al*.^9^ except that melting curve detection was performed at 95 °C for 5 sec; 60 °C for 1 min (please see supplementary material for the details of conditions applied, and Table S2 for sequences of the Real-Time primers used). The data were analyzed using the comparative 2^−∆∆^CT^69^ considering *Act-5c* as the internal control. Differences in the fold change of transcript levels between groups were analyzed statistically by employing One-way Analysis of Variance (ANOVA).

### Immunofluorescence and confocal microscopy

The testes/gonads from control and knockdown (ERR-TRiP) males/larvae, respectively, were dissected in physiological saline, and transferred separately to phosphate buffer saline (PBS), with 0.3% Triton-X (PBS with Triton-X, referred to as PBX). At the end of dissections, PBX was replaced, and the tissues were incubated in 4% paraformaldehyde for 1 h, followed by three washes with 0.3% PBX, for 15 min each. The tissues were then kept immersed in blocking solution (3% bovine serum albumin, BSA in 0.1% PBX) for 30 min. Subsequently, adult tissues were incubated overnight in primary antibodies against bam, vasa, eya or alpha spectrin while larval gonads were incubated overnight in primary antibodies against Sox100B at 4 °C (for additional details of the employed protocol and name/host/dilutions of the primary antibodies used, please refer to supplementary material, and Table S3). The tissues were then washed thrice, for 15 min each with 0.3% PBX incubated at room temperature for 2 h in secondary antibody (please refer to supplementary methods for details of secondary antibodies used). Tissues were washed prior to mounting in vectashield (Vector labs, USA). Nuclei were stained with DAPI (pre-incorporated in the vectashield) and visualized under confocal microscope (TCS SPE, Leica, Germany; please refer to supplementary methods for settings/excitation wavelengths). A minimum of five independent immunostaining batches, each having at least 5 replicates (pairs of testes/gonads) were analyzed in each case.

### Analysis of mature sperm, Protamine incorporation into sperm chromatin in control/knockdown males and sperm storage in their mates

To determine if ERR mediated effects on spermatogenesis are reflected at the level of sperm, we examined the Protamine incorporation into sperm chromatin and also counted the mature sperm in seminal vesicles of control and knockdown males three days post eclosion. Briefly, ProtB-EGFP/ProtB-EGFP; testes-GAL4/Sb females were crossed with UAS-ERR TRiP males to generate knockdown/control males carrying ProtB-EGFP tagged sperm. We examined Protamine incorporation into sperm chromatin by looking for the presence of EGFP labelled sperm heads in control/knockdown testes and seminal vesicles under the fluorescence microscope (at a total magnification of 600X, with GFP filter; Leica DMLB, Germany). Subsequently, the number of mature sperm stored in seminal vesicles of control/knockdown males were counted by using EGFP tag. Later, to determine the storage of sperm from control/knockdown males in the reproductive tracts of their mates, sperm in the sperm storage organs (seminal receptacle and paired spermathecae) of females mated to control/knockdown males, 2 h ASM were counted as in Misra *et al*.^9^. Mature sperm in seminal vesicles of control/knockdown males and in sperm storage organs (seminal receptacle and spermathecae) of their mates were counted twice. Every group contained 15–20 replicates, and the differences, if any, in above parameters, in the respective groups were analyzed statistically through Mann-Whitney U test.

### Transmission electron microscopy of adult/pupal Drosophila testes

The testes dissected from the control and knockdown males from both ERR lines (ERR-miRNA & ERR-TRiP) and those dissected from pupae of Sox100B-TRiP lines were incubated, independently, in fixing solution (4% Paraformaldehyde, 2% Glutaraldehyde, 0.05% Sucrose, and 0.1% Sodium Cacodylate) for 90 min. The tissues were washed thrice in 0.1 M Sodium Cacodylate for 20 min each, prior to overnight incubation in 1% Osmium tetraoxide (OsO_4_). The tissues were then washed thrice in 0.1% Sodium cacodylate for 20 min each and were treated with 1% OsO_4_ for 2 h. These tissues were sequentially dehydrated in 15%, 30%, 60% and 100% acetone, respectively, for 20 min. Subsequently, tissues were embedded in a low viscosity epoxy resin araldite medium to prepare blocks. These blocks were used to cut semi and ultrathin sections using a ultra microtome (EM-UC7 ultramicrotome, Leica, Germany). Sections were stained in Toluidine blue staining solution. The sections were examined in a Tecnai G2 spirit TWIN transmission electron microscope (FEI, USA), equipped with digital CCD camera (Gatan, Netherland, Europe) and the structural details of testicular sections were recorded (1 μm, 15000X, and 0.5 μm, 30000X). The electron microscopic analysis was performed thrice per batch (control/ERR-miRNA/ERR-TRiP/Sox100B-TRiP) with each batch having a minimum of five replicates (pairs of testes).

## Additional Information

**How to cite this article**: Misra, S. *et al*. Estrogen related receptor is required for the testicular development and for the normal sperm axoneme/mitochondrial derivatives in *Drosophila* males. *Sci. Rep.* **7**, 40372; doi: 10.1038/srep40372 (2017).

**Publisher's note:** Springer Nature remains neutral with regard to jurisdictional claims in published maps and institutional affiliations.

## Supplementary Material

Supplementary Information
