# Supplementary material for: Estrogen related receptor is required for the testicular development and for the normal sperm axoneme/mitochondrial derivatives in Drosophila males
Source: Sci Rep. 2017 Jan 17;7:40372. doi: 10.1038/srep40372 (PMC5240334; doi:10.1038/srep40372)
Supplement: Supplementary Information [file srep40372-s1.pdf]

Estrogen related receptor is required for the testicular development and for the normal sperm axoneme/mitochondrial derivatives in *Drosophila* males

Snigdha Misra[1,2], Anuj Kumar Pandey[1], Snigdha Gupta[1,2], Ajay Kumar[1,2], Priyanka Khanna[1], Jai shankar[3], Kristipati Ravi Ram [1,2§]

[1]Embryotoxicology Laboratory, Environmental Toxicology Group, CSIR-Indian Institute of Toxicology Research (CSIR-IITR), MG Marg, Lucknow, Uttar Pradesh, 226001, India. [2] Academy of Scientific and Innovative Research (AcSIR), CSIR-IITR campus, Lucknow, Uttar Pradesh, 226001, India. [3]Electron Microscopy Facility, CSIR-Indian Institute of Toxicology Research (CSIR-IITR), MG Marg, Lucknow, Uttar Pradesh, 226001, India.

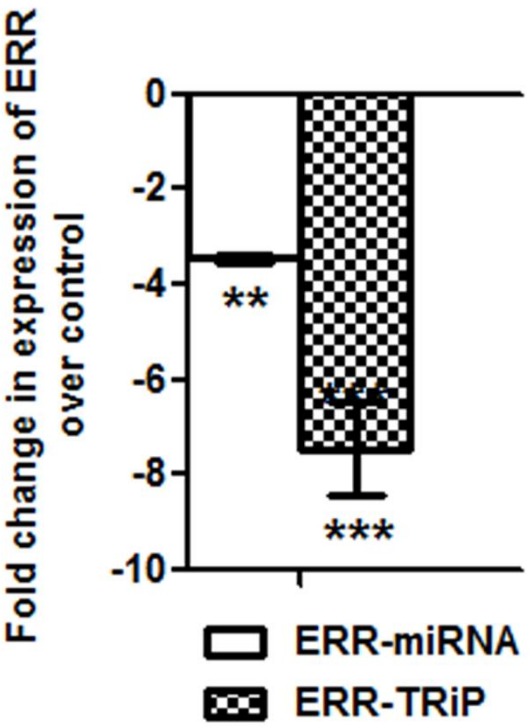

Figure S1. Confirmation and determination of the extent of knockdown of ERR through quantitative real time PCR based analysis of ERR transcript levels. The levels of ERR transcripts were reduced to 3.5 folds in miRNA based knockdown driven by testes-GAL4 (*$p<0.05$, represented as ERR-miRNA), while 7.5 folds reduction was observed in knockdown males produced from UAS-ERR-TRiP (**$p<0.01$, represented as ERR-TRiP), when compared to their controls. The Δct values were determined through normalization against Act-5c, which was used as an internal control for the quality of the template.

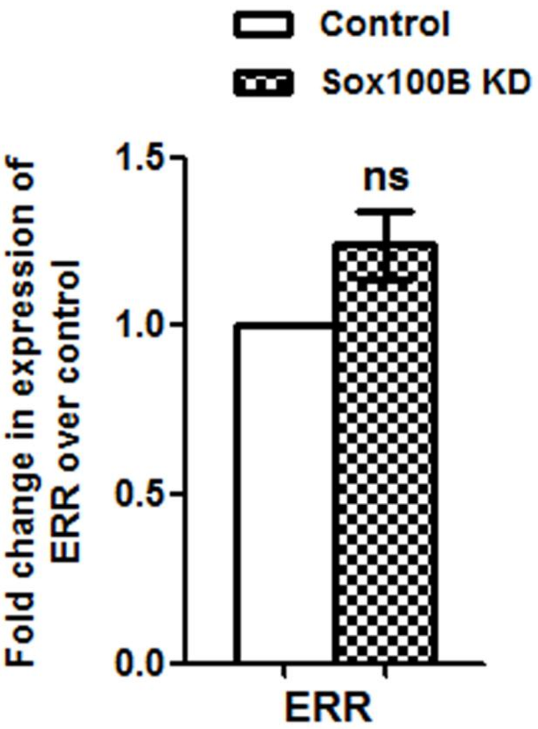

Figure. S2. Quantitative real time PCR based analysis of ERR transcript levels in males with systemic knockdown of Sox100B. The levels of ERR transcripts in male pupae where Sox100B dsRNAi was driven through Act5c-GAL4/ Tb were comparable to control. The Δct values were determined through normalization against Act-5c, which was used as an internal control for the quality of the template.

Supplementary methods

Table S1. Expression patterns of the GAL4s used

Stock No.	Expression Pattern
BL12608	Late spermatogonia, early spermatocytes, cyst cells, pigment cells, muscle sheath
BL13134	Early spermatocytes, cyst cells, muscle sheath
BL12772	Transient in early spermatocytes, cyst cells, pigment cells, muscle sheath
BL6983	Adult male accessory gland, testes sheath, cyst cells, larval salivary gland, and adult female columnar follicle cells
BL6987	Adult ovary, amnioserosa, adult male accessory glands and seminal vesicles.
BL6989	Accessory glands, cyst cells and germ cells
BL1947	Male accessory glands
Bam-Gal4	Germ cell/ Spermatogonia
Tj-Gal4	Early cyst cells
Nos-Gal4	Early germline cells
Act5c-GAL4	Systemic expression

Conditions applied for Real time PCR

1 cycle of pre incubation (50ºC for 2 min and 95ºC for 10 min) followed by 40 amplification cycles (95ºC for 15 seconds, and 60ºC for 1 min) and melting curve detection (95ºC for 5 sec, 60ºC for 1 min). Experiments were preformed in duplicate for each sample for ten genes (please see Table S2 for primer sequences). The gene expression data were analyzed using the comparative $2^{-\Delta\Delta}$CT[1] considering Act5c as the internal control. All results are given as Mean±Standard Error of the mean.

Table S2. Primers used for Real time PCR

Gene	Primer Sequences
Armadillo (*arm*)	GATGAGGATCAGGTGGTAGTTTC<br>CCATCTGAGGGCTGTTCATAAT
hopscotch (*hop*)	GACATACCCTTTCTACCCTTTCC<br>CCCTTGTAGACAGTGCCATAAT
always early (*aly*)	AATCTCCAGCGTCGCTATTC<br>TCCTCGTCCTGCTTCTGATA
meiotic arrest (*mia*)	GCGTTCTCTATCCGCGATATT<br>CCAGCCTCGTTTAGCAGATT
Bruce	CAGATGGCACCGTGGAAATA<br>CCTATCCAAGTTCCGGCAATAA
fuzzy onions (*fzo*)	GTCCTTCAATGTCTCTCCATACC<br>CAATCCAGGCCGTAGATTAGTT
canonball (*can*)	ACACGTCGCAATCTCTTACTT<br>CTCCTCCTTGTGCTTGTTCT

bag of marbles (*bam*)	CTGTTCATCGCCCAGAGATAC<br>CGGGAAATAGGTCTCTGGATTG
benign gonial cell neoplasm (*bgcn*)	CTATGCCGTGGACACTAGAAAG<br>GGAAGCTCTGTGCCGAAATA
Actin (*Act-5c*)	CGATTTGACCGACTACCTGATG<br>GCACAGCTTCTCCTTGATGT
Sox100b	AGGGTCATGTCCAAACAGTATC<br>GGGCTTCTTATCACTGTCCTTTA
ERR	CACAGCGCATGGAAAGAATATC<br>GAACTCTGATCATCCAGCAGAA

Immunoflorescence and confocal microscopy

The testes/ gonads from the male reproductive tract of control and knockdown males were dissected in physiological saline, and transferred separately to phosphate buffer saline (PBS), with 0.3% Triton-X (PBS with Triton-X, referred to as PBX). At the end of dissections, PBX was replaced with 4% paraformaldehyde prepared in 1x PBS, and the tissues were incubated for 1h. The tissues were then washed with 0.3% PBX thrice, for 15min each. The blocking solution comprising 3% bovine serum albumin (BSA) in 0.1% PBX was added, and tissues were incubated for 30min. The primary antibody was added, and tissues were incubated overnight at 4ºC (For details of the name/ host/ dilutions of the primary antibodies used, please refer to Table S3). Subsequently, tissues were washed thrice, for 15min each with 0.3% PBX, and incubated at room temperature for 2h in secondary antibody (Alexa Fluor® 488 Rabbit Anti-Mouse; Alexa Fluor® 488 Goat Anti-Rabbit, Life technologies, USA), diluted in 0.1% PBX at 1:200 folds in dark. Tissues were washed thrice, for 15min each with 0.3% PBX prior to mounting them in vectashield (Vector labs, USA). Nuclei were stained with DAPI, which is pre-incorporated in the vectashield. The fluorescence was visualized under confocal microscope (TCS SPE, Leica, Germany), using in-built settings for the DAPI (excitation wavelength 340-380nm), and FITC (excitation wavelength 450-490nm). A minimum of five independent immunostaining batches, each having at least 5 replicates (pairs of testes) were analyzed in each case.

Table S3. Primary antibodies used in this study

**Antibody / Cat. No**	**Host**	**Dilution**	**References**
bam-c<br>DSHB-C1-252	Mouse	1:10	[2]
vasa	Rat	1:40	[3]
eya10H6-c<br>DSHB-C1-385	Mouse	1:25	[4]
3A9 (323 or M10-2)-c (α-spectrin)<br>DSHB-C1-86	Mouse	1:25	[5]
Anti-Sox100B<br>AAS77513C	Rabbit	1:25 for Immunofluorescence<br>1:1000 for western	M/s Antibody Verify, USA

1. Schmittgen, T. D. & Livak, K. J. Analyzing real-time PCR data by the comparative C(T) method. *Nat. Protoc.* **3**, 1101-1108 (2008).

2. McKearin, D. M. & Spradling, A. C. bag-of-marbles: a Drosophila gene required to initiate both male and female gametogenesis. *Genes Dev.* **4**, 2242-2251 (1990).

3. Renault, A. D. vasa is expressed in somatic cells of the embryonic gonad in a sex-specific manner in Drosophila melanogaster. *Biol. Open* **1**, 1043-1048 (2012).

4. Bonini, N. M., Leiserson, W. M. & Benzer, S. The eyes absent gene: genetic control of cell survival and differentiation in the developing Drosophila eye. *Cell* **72**, 379-395 (1993).

5. Dubreuil, R. R., Maddux, P. B., Grushko, T. A. & MacVicar, G. R. Segregation of two spectrin isoforms: polarized membrane-binding sites direct polarized membrane skeleton assembly. *Mol. Biol. Cell* **8**, 1933-1942 (1997).
